# Machine Learning in Point of Care Ultrasound 

**DOI:** 10.24908/pocus.v7iKidney.15345

**Published:** 2022-02-01

**Authors:** Momodou L Sonko, T Campbell Arnold, Ivan A Kuznetsov

**Affiliations:** 1 The Perelman School of Medicine, The University of Pennsylvania

**Keywords:** POCUS, point of care ultrasound, machine learning

## What are Machine learning and Deep Learning?

When a patient presents to the ED, clinicians often turn to medical imaging to better understand their condition. Traditionally, imaging is collected from the patient and interpreted by a radiologist remotely. However, scanning devices are increasingly equipped with analytical software that can provide quantitative assessments at the patient’s bedside. These assessments often rely on machine learning algorithms as a means of interpreting medical images.

A machine learning (ML) algorithm is able to utilize presented data to adapt and learn without following explicit instructions. ML is a branch of artificial intelligence (AI) and has garnered a great deal of attention over the past decade, due in large part to substantial advancements in data processing and improvements in model performance. ML has proven to be a powerful method for interpreting complex data. Clinicians may understand all the information necessary to classify a patient’s condition, but seldom can they derive an equation that communicates precisely what information is relevant and irrelevant. ML excels at this task and permits scientists to develop solutions without knowing how to explicitly code the answer. In the most common form of ML, called supervised learning, scientists provide data inputs (called features) and corresponding class labels. The machine learning algorithm then determines what input features are relevant to predict the class labels, thus generating a model that can take in novel features and provide a predicted class label as output.

One of the most successful methods for solving medical imaging problems is a subfield of machine learning called deep learning (DL). Deep learning was inspired by the complex neural architecture of the human brain, which is organized into interconnected layers of neurons and can solve incredibly complex problems. In the primate visual cortex, simple photoreceptor input is passed through convolutional layers in the ventral visual stream of the brain. Each successive layer produces increasingly complex representations of the photoreceptor input, which permits humans to classify the objects and interpret the scenes they see. Similarly, deep learning algorithms simulate the ventral visual stream by passing image information through multiple layers of a convolutional neural network (CNN). These networks process simple pixel information, form new complex representations, and pass those representations on to subsequent layers for eventual image classification 1. 

**Figure 1:**
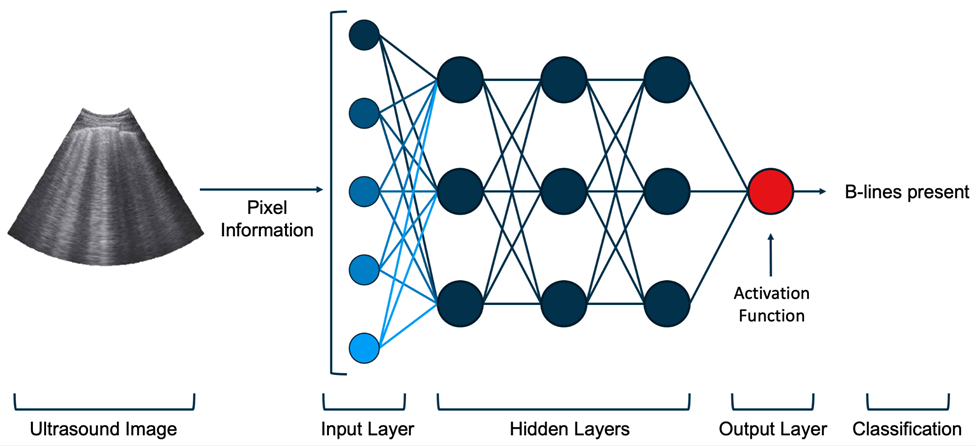
Example convolutional neural network for ultrasound. Here we present a simplified network architecture for a model designed to detect the presence or absence of B-lines in a lung ultrasound image. The model takes in raw data, in this case pixels from the lung ultrasound image. Neurons in the input layer, represented here by circles, are activated to varying degrees by the pixels. These activations are passed forward through several convolutions in the hidden layers. Final activation values are fed into the activation function in the output layer. The output is a binary classification: either presence or absence of B-lines.

To train a CNN to classify images, pixel values are passed into the initial layer of neurons which are activated by the information (Figure 1). The activations are then fed forward into additional hidden layers which further process the data. The features generated by this process are fed through a final activation function, which provides a classification label in the output layer. To improve model accuracy, algorithm predictions are compared to provided labels. A cost function assesses the difference between model predictions and actual values, awarding a proportional penalty to the model. The goal of the training process is to minimize the cost or penalty awarded to the model. Using an optimization technique called gradient descent, the response weight of each individual neuron in the network is iteratively tuned such that the final classifications better match the expected output, thus reducing the penalty assessed by the cost function. After training on a corpus of images, a novel image can be fed into the algorithm and a predicted classification will be output.

Over the past several years there have been huge advances in the use of ML and DL algorithms to address a number of challenges clinically. DL algorithms have been extensively used within the field of radiology where they are used to perform numerous tasks, including segmentation of anatomical structures or local lesions, detection of probable tumors, and classification of lung and breast nodules 2, 3. A prominent example of how DL is rapidly changing the field of radiology can be seen in chest X-ray advancements. In 2017, with the release of the world's largest publicly available chest X-ray dataset (over 100,000 frontal-view X-ray images) by Stanford and the NIH 4, P. Rajpurkar et al. developed a DL system called CheXNet that could automatically detect and classify 14 different diseases on chest X-ray 5, 6. While there are some concerns surrounding the validity of human to algorithm comparisons 7, their system was able to achieve a comparable detection rate to expert radiologists for most diseases and demonstrated the promise of DL systems within medical imaging 8.

## Challenges to greater adoption of DL in POCUS

The example above demonstrates the potential of ML systems to improve clinical care for patients as well as assist radiologists with their clinical workload. However, despite rapid advancements in many medical imaging modalities, similar applications of ML algorithms to point of care ultrasound (POCUS) have been slower to arrive. This discrepancy is present for a number of reasons. First, unlike POCUS imaging modalities such as chest X-ray, CT, and MRI, have standardized imaging protocols. Hospital image archiving infrastructure was designed to store and save imaging data for later use. As a result of the persistent imaging infrastructure for these modalities large, organized imaging datasets have been developed that can be more readily interrogated by DL algorithms. 

In contrast, images and video acquired at the bedside using POCUS are often used for immediate physician support and not always permanently archived for later analysis. Additionally, the point of care setting inherently introduces variability in data quality even when collected by the same sonographer. Variation in sonographer skill level, image acquisition order, and technique further complicates ultrasound datasets. Even in well-performed scans, imaging distortion and artifacts are often an inescapable reality for POCUS. This results in ultrasound images containing a great deal of “noise” or randomness in the data. Variability is further compounded when combining images from different scanner manufacturers or academic centers into a single dataset. Additionally, ultrasound images often lack global reference structures, making it difficult to determine exactly where on a patient’s body an image was collected. Finally, as an imaging modality, POCUS is relatively new compared to chest X-ray, CT, and MRI, only achieving widespread use in hospitals in the 1990s 9. Taken together, these reasons explain why there are relatively few DL applications for POCUS compared to other imaging modalities.

Nonetheless, the last few years have seen an explosion of novel DL applications within POCUS. DL is uniquely suited for analysis of POCUS because it is able to generate high-level abstractions from a wide array of raw imaging data of varying quality. This ability to “cut through the noise” and draw abstractions and note otherwise missed patterns has been one factor leading to greater use of DL within POCUS. Increased interest in DL has come in part due to unique computational approaches to address the obstacles previously mentioned. Both traditional machine learning techniques (i.e. random Forest classifiers, support vector machines) and deep learning methods (i.e recurrent neural networks (RNN), auto-encoders), have been employed on ultrasound datasets with good success. Additionally, researchers have utilized innovative techniques such as transfer learning to circumvent some issues related to limited and inconsistent datasets. Transfer learning is the process of initializing a DL model with weights derived from another training task and fine-tuning the model to perform a new task with the goal of reducing the number of trials necessary to learn a similar task 10, 11. For instance, a model trained to accurately identify and segment straight lines may be retrained on a carotid ultrasound dataset in order to identify and segment the arterial wall. This approach has the benefit of generally requiring fewer class labels in the training set in order to develop a successful algorithm. 

DL algorithms have the potential to further increase the utility and adoption of POCUS. Many important uses of ML applied to POCUS are outside the scope of this review, but also include DL algorithms applied to enable novice sonographers in acquiring the best image^12^ and as educational tools for medical students and residents providing procedural training on needle guidance for epidural anesthesia 13. Herein we will discuss potential and emerging clinical uses of ML approaches applied to POCUS. 

## Current Clinical Applications of ML within Ultrasound

Here we will briefly highlight some of the clinical ML algorithms that have been developed for POCUS. To date, there are relatively few real-time ML algorithms available in POCUS (Table 1). With the exception of a few commercially available models, the majority of ML algorithms were developed for US applications using datasets captured from retrospective studies. One barrier to greater adoption of ML models within POCUS is the need to implement software in real-time on the ultrasound device hardware itself. Adoption of ML in POCUS is challenging not only because software and hardware must be integrated to enable real time applications, but also because most recently developed ML models lack sufficient clinical validation and FDA approval to be used in the clinical setting. While this regulatory milestone may seem distant, it has already been achieved for similar medical imaging applications. In 2018 the FDA approved a retinal imaging device with onboard artificial intelligence that could make diagnostic decisions, a first of its kind innovation 14. As researchers continue to develop ML models for ultrasound, it is important to note that given adequate implementation, many of these models can be adapted for POCUS devices in the near future.

**Table 1:** Discussed studies on the application of machine learning in ultrasound. Due to differences in study design, inter-study performance cannot be compared. Refer to original studies for details of study design. Acronyms: ED - end diastolic; ES - end systolic; RNN - recurrent neural network; LSTM - long short-term memory network; CNN - convolutional neural network; DSC - Dice score coefficient; EF - ejection fraction; LV - left ventricle; FASP - fetal abdominal standard plane; FFASP - fetal face axial standard plane; FFVSP - fetal four-chamber view standard plane; CKD - chronic kidney disease; DVT - deep venous thrombosis; ICC - interclass correlation coefficient; CI - confidence interval. (con’t next page…)

**Authors**	**Application**	**Model**	**Performance**
Dezaki et al.	Cadiac: cycle phase determination (ED vs ES)	Residual RNNs (ResNet + LSTM)	R² score = 0.66 Error ED = 3.7 Error ES = 4.1
Smistad et al.	Cardiac: LV segmentation	CNN (U-Net 15)	DSC = 0.86 ± 0.06
S. Chen et al.	Cardiac: plane detection, LV detection, LV segmentation	PSPNet + temporal affine network (TAN)	DSC = 0.91
Knackstedt et al.	Cardiac: EF	AutoLV	ICC = 0.70-0.83
Thavendiranathan et al.	Cardiac: LV volume & EF	Probabilistic contouring algorithm composed of a Bayesian framework, hierarchical K-means clustering, and probabilistic boosting tree.	Correlation with cardiac magnetic resonance measurements: ED volume = 0.90 ES volume = 0.96 EF = 0.98
F. Dominika et al.	Cardiac: EF	LVivo EF (DiA Imaging Analysis)	Performance relative to calculated EF via 3D echocardiography: Pearson correlation = 0.92 (95% CI 0.87-0.95) Mean difference = 0.61% (95% CI -0.68-1.89%)
Nafee et al.	Extremities: DVT	Ensemble classifier	Concordance statistic = 0.69
Tanno et al.	Extremities: DVT	CNN	F1 score = 90%
H. Chen et al.	Fetal: plane detection (FASP, FFASP, FFVSP)	Transferred RNN (T-RNN): CNN + LSTM	Accuracy: FASP = 0.91 FFASP = 0.87 FFVSP = 0.87
Jang et al.	Fetal: abdominal circumference	CNN + U-Net	Accuracy = 87.1%
Gao et al.	Fetal: anatomy classification	T-CNN	Accuracy = 91.5%
Ravishankar et al.	Kidney: segmentation	Ensemble classifier with gradient boosting	DSC = 0.83
Wu et al.	Kidney: segmentation	Cascaded DenseNet	Mean intersection over union = 0.83
C. Chen et al.	Kidney: CKD detection	Support vector machines	5 stages: Accuracy = 70%
Kuo et al.	Kidney: CKD detection	ResNet	5 stages: Accuracy = 85.6%
Christiana et al.	Lung: B-line score	Custom shallow CNN (10 layers)	Presence vs absence: Sensitivity = 93% Specificity = 96% Severity (0-4): Kappa = 0.65
Sonko et al.	Lung: B-line score	Autoencoder + CNN	Presence vs absence: Accuracy = 87.3% Score (0-4): Accuracy = 60.5%
Correa et al.	Lung: pneumonia	Custom feedforward neural network (3 layers)	Sensitivity = 91% Specificity = 100%
Born et al.	Lung: COVID detection	CNN (VGG-16)	Sensitivity = 0.96 Specificity = 0.79
J. Short et al.	Lung: auto mated B-line counting	Auto B-lines (GE Healthcare Venue Go)	Correlation with expert interpretation: ICC = 0.794 (95% CI 0.736-0.840)

As mentioned before, the majority of new ML algorithms within US have been applied using DL architectures. Some of the most significant advances in DL applied to POCUS have taken place within echocardiography. Here, a number of models have been developed for a wide number of classification, segmentation, and detection tasks. A frequently used DL application involving both segmentation and biometric measurements has been the rapid determination of cardiac ejection fraction (EF). In order to accurately determine EF using echocardiography, determination of cardiac cycles–namely, end-diastole and end-systole–is necessary. Some groups such as Dezaki et al. have successfully used ML models to accurately determine cardiac cycles^16^ and a number of other groups have also successfully trained DL models to segment various chambers of the heart using recurrent neural networks (RNN) and CNNs 17, 18, 19. Furthermore, the automation of EF and cardiac volumes using ML has been shown to have excellent agreement between automated and manual approaches, with increased efficiency and reproducibility of measurements 20, 21.

There has also been significant interest in applying ML algorithms to lung ultrasound. Lung ultrasound has gained increased use in the POC setting due to the wide number of clinically useful assessments it provides 22. The quantitative assessment of B-line score (BLS) has become an important tool for assessing pulmonary congestion using POCUS 23. B-lines are hyperechoic reverberation artifacts arising from the pleural surface that extend to the bottom of the screen without fading and move in tandem with lung sliding. Total BLS can be used to determine fluid overload (FO) severity score and a number of studies have demonstrated that BLS accurately quantifies pulmonary congestion outperforming the physical exam and chest x-ray 24, 25, 26. Additionally, in the point of care setting, rapid assessment of a patient’s volume status can be a crucial tool in guiding clinical interventions. Yet, widespread use of this technique is limited partly due to the tedious nature of the assessment.

A number of groups, including our own, have developed DL models using CNN to automatically quantify B-line scores from POC lung ultrasound video clips. Recently, B. Christiana et al. developed a supervised CNN trained on 400 lung ultrasound clips to calculate total BLS in emergency department patients. They achieved a binary classification (B-lines present versus absent) sensitivity and specificity of 93% and 96% compared to an expert interpreter. In multiclass classification of B-line severity their DL model achieved a linear weighted kappa of 0.65 vs an interrater reliability of 0.87 27. Our own group has developed a DL model that uses a transformer block architecture CNN trained on 91 hemodialysis patients with ESRD to calculate total BLS and severity level. In preliminary results, our DL model demonstrated a binary classification (presence versus absence of B-lines) accuracy of 87.3% and a total BLS classification (scored 0-4) accuracy of 60.5% 28. 

Point of care lung ultrasound has also shown great promise in the accurate diagnosing of community acquired pneumonia (CAP). It has demonstrated excellent diagnostic capabilities when performed by a trained sonographer compared to both clinical assessment and chest X-ray, while also avoiding unnecessary radiation exposure in vulnerable patients such as pediatric populations 29, 30, 31. However, lung ultrasound is not included in the diagnostic workup for CAP partly because of inter-operator variability of lung ultrasound and training. DL approaches aimed at reducing these barriers are another emerging trend. In 2018, Correa et al. developed a neural network trained on 1450 CAP-positive ultrasound frames from a hospitalized pediatric population in Peru. The algorithm was successful in correctly identifying pneumonia infiltrates with 90.9% sensitivity and 100% specificity 32. 

More recently, amidst the 2020 COVID-19 pandemic, there has been increased attention paid to increasing the diagnostic ability of clinicians to detect the presence of the novel coronavirus in patients. To this end, Born et al. developed a deep CNN trained on over 1100 COVID-19 confirmed lung ultrasound images to achieve a detection sensitivity of 0.96 and specificity of 0.79 and F1-score of 0.92 in a 5-fold cross validation 33. The authors provide an open-access web service (POCOVIDScreen) that deploys the predictive model, allowing clinicians to both perform predictions on ultrasound lung images and upload their captured images to add to the database.

Use of POCUS within nephrology has also increased in use over the past several years. In patients with chronic kidney disease (CKD), volume overload plays an important role in the disease pathology by complicating cardiovascular pathophysiology leading to increased cardiovascular morbidity and overall mortality 34, 35. For patients with end-stage renal disease (ESRD) on hemodialysis, it has also been shown that the extent of volume overload correlates with adverse cardiovascular events 36. Therefore, for the nephrologist, close monitoring of their patient’s overall volume status is important in the clinical management of patients. Thus, POC lung ultrasound (and BLS quantification) has also become an important tool in the nephrologist’s arsenal. However, other ML advancements within renal ultrasound include the accurate segmentation of the kidneys, yielding rapid and accurate measurement of renal dimensions in patients by groups such as Ravishankar et al. and Wu et al. 37, 38, as well as the detection of various stages of CKD by groups such as Chen et al. and Kuo et al. 39, 40.

Fetal measurement is another widely used application in POCUS. In the emergency room, rapid and accurate assessment of fetal parameters such as crown-rump length and classification of the abdominal standard plane, are important to avoid misdiagnosis and guide appropriate interventions 41. Several groups have developed deep learning models for fetal exams. In a two-step process, Jang et al. first developed a CNN to identify the abdominal standard plane and then trained a model to segment and estimate fetal abdominal circumference from fetal ultrasound images 42, 43. Gao et al. developed a CNN that categorized abdominal freehand sweep images into four categories: fetal abdomen, heart, skull, or other. They trained two models, one using only obstetric ultrasound images and a second that employed transfer learning, using a pretrained ImageNet model and fine-tuning it on obstetric ultrasound images. Transfer learning improved classification accuracy in all categories of fetal anatomical structures compared to their non-transfer learning approach 44.

The final clinical application of ML for POCUS discussed here is deep-venous thrombosis (DVT) screening. POCUS is an important tool for physicians treating potential DVT patients within the emergency room as well as in the inpatient setting. POCUS can guide clinical decision-making for patients at risk for, or suspected of having, a pulmonary embolism 45. During the exam, the deep veins of the lower extremity are compressed along their course and areas of low compressibility suggest potential thrombus formation at that location. Recently, Nafee et al. sought to evaluate the performance of two ML models they developed versus a validated DVT scoring system in acutely ill patients. Their study demonstrated that both of their ML apporaches outperformed the validated manual scoring system in predicting venous thromboembolism (VTE) (c-statistic: ML methods = 0.69 and 0.68, manual scoring system = 0.59) 46. 

Other models such as that by Tanno et al. have aimed to increase classification accuracy of DVT scans by automatically detecting the extent of vein compressibility in DVT scans 47. Researchers proposed a dual-task CNN to predict vein compressibility with an F1 score of 90% when evaluated on 1150 5–10 s compression image sequences from 115 healthy volunteers resulting in a data set size of approximately 200k labelled images. As further development continues, these advancements may greatly increase the accessibility and clinical usage of this already impactful diagnostic study.

## Commercially Available products utilizing ML

Of note, companies such as Mindray and GE have utilized ML and DL based algorithms in commercially available echocardiography products to perform automated tasks such as automated EF calculation, LV border identification, and chamber length calculations (Mindray North America, Mahwah NJ; GE Healthcare, Chicago IL). Newer devices entering the market are now often branded with “AI enabled” capabilities, such as left ventricular outflow tract (LVOT) plane identification and Doppler placement (see: GE Venue and Mindray). These tools are beginning to make their way into newer POCUS devices as well. Companies like Butterfly Network, Bay Labs, and Clarius have released POCUS probes that contain AI-enabled cardiac algorithms for automated EF estimation as well as cardiac chamber segmentation (i.e. Butterfly Network’s IQ probe). 

There is also commercial interest and new adoption of automated B-line counting algorithms within POCUS. Notably, GE has incorporated an auto B-line counter within their new suite of GE Venue Go POCUS devices. Their model uses computer vision and DL approaches, including a proprietary CNN, to automatically detect and count B-lines in lung ultrasound scans. A study by J. Short et al. found that automatic counting of lung B-lines was consistent with visual counting, as performed by experts in the field and both systems showed a high intra- and interobserver reliability 48. Other device manufacturers such as Mindray have similarly developed their own automatic B-line counting algorithms using a mixture of traditional computer vision systems and DL approaches.

Interestingly, the clinical ML software market has grown to now support firms whose business models almost entirely center around developing novel algorithms for clinical use intended for device manufacturers. DiA Imaging Analysis Ltd. is one notable firm in this category. They partner with ultrasound device manufacturers and large academic medical centers to develop AI-enabled solutions for ultrasound. Currently, as mentioned previously, much of the development for these solutions has focused on POC echocardiography, but additional interest has been shown in the development of AI-enabled abdominal algorithms as well. The company has additionally partnered with GE to offer the first AI-based solution for automated EF analysis on handheld ultrasound through the “LVivo EF” on GE’s Vscan Extend, which has been shown to yield similar EF values as 3D echocardiography 49. As interest in DL applications within POCUS continues to blossom, it is likely that additional firms similar to DiA will emerge, outsourcing much of the ML innovation once developed in-house by ultrasound device manufacturers to specialized image analysis companies.

## Future Steps & Upcoming Advancements

The dynamic and real-time nature of POCUS provides a major advantage over other imaging modalities such as CT and MRI. Yet, this also represents a major challenge for researchers developing ML algorithms for POCUS. A trained sonographer will rarely examine a single image frame to make a clinical assessment of a patient; rather, data from multiple frames are assessed simultaneously together to inform the clinician of a proper course of action. Within the broader context of deep learning, a known issue is that most state-of-the-art architectures are optimized for single image classification and that impressive performance does not necessarily generalize to video-type data, such as POCUS. 

A variety of methods have been applied to try to generalize methods used for image classification to video classification. Perhaps the most direct implementation of this has been the use of 3D CNNs (as opposed to the 2D ones used for single image classification). For example, Hara et al. extended the state-of-the-art ResNet architecture to 3D by adjusting the original 3x3 kernels to 3x3x3 50. However, introducing 3D convolutions leads to significantly increased computational overhead and increases network complexity, hence yielding longer training times and increased likelihood of overfitting models 51. Progress on this front has been made by mixing 2D and 3D convolutions and using R(2+1)D convolutions (wherein 3D convolutions are factorized into spatial and temporal convolutions) 52, 53. Such architectures show great promise for application in POCUS, but the complexity of such networks leads to requirements for large amounts of data, which are often unavailable.

Another approach, first proposed by Simonyan et al., involves processing video data as two separate streams: a spatial and temporal stream 54. The spatial stream is designed to classify still video frames and typically consists of a 3D CNN or a conventional 2D CNN which sequentially processes frames. The temporal stream is meant to capture inter-frame changes and is created by combining optical flow data from several frames. Generally, these two-stream CNNs outperform both conventional 2D and 3D CNNs for video classification. Howard et al. applied such a two-stream CNN to automatically determine the scan view from echocardiography data. Such two-stream CNNs can potentially lower the computational overhead for POCUS analysis and classification 55 **.**

A major area of interest in our group and others has been the application of attention-gated networks to DL ultrasound. Attention mechanisms attempt to better mimic human perception by using surrounding local information in the data to contextualize a specific target. Attention models have been heavily used for natural language processing (NLP) tasks, where integrating information from potentially distant parts of a sentence is necessary to accurately translate a given word 56. Here, transformer block architectures have been used with success 57. Attention mechanisms were first used by Mnih et al. in a recurrent neural network (RNN) for image classification 58, but has since been applied to a variety of ultrasound image analysis including in fetal ultrasound scan plane detection 59. Attention models could prove useful in a variety of POCUS models including B-line score (BLS) determination from lung ultrasound. Accurate determination of BLS often depends on assessing adjacent frames rather than relying on a single frame. Attention models have the additional advantage of giving insight into which video time frames and what image content the algorithm is attending to for deriving its classification, thereby potentially improving interpretability.

Additionally, researchers have developed alternative approaches to identifying optimal network architectures through neural architecture search. Generally, network architectures are designed by data scientists using some a priori hypothesis of underlying data structure. This time-consuming task leaves the entirety of alternative network architectures largely unexplored. To address this issue, scientists and Google’s AI division developed a neural architecture search, where machine learning techniques are used to optimize the network architecture while training the network itself 60, 61. This approach has been successful for improving the architecture of conventional (image-classification) CNNs and is now being applied to video CNNs. Piergiovanni et al. designed EvaNet, wherein they used an evolutionary algorithm to explore different layer types and combinations that could optimally represent the relationships between spatial and temporal aspects of videos 62. Ryoo et al. designed AssembleNet, a network composed of multiple sub-network blocks that interprets input videos as multiple input streams sampled at different levels of temporal resolution 63. AssembleNet is able to optimize the connectivity between both the sub-network blocks as well as the connectivity between the multiple variable-resolution streams. Such techniques are already being applied for medical image analysis. For example, Yan et al. developed MS-NAS (Multi-Scale Neural Architecture Search for Medical Image Segmentation) and applied it to outperform several state-of-the-art algorithms used for segmentation of CT images 64. Given the temporal dynamics and acquisition complexities of ultrasound data, a priori hypotheses are unlikely to arrive at efficient network structures. Neural architecture search techniques, such as MS-NAS, will permit data-driven approaches to developing optimized algorithms that can address the broad range of ultrasound image processing problems faced by clinicians.

## Conclusion

In conclusion, we have introduced the concepts of machine learning and deep learning, reviewed current applications of these powerful tools in POCUS, discussed available commercial products utilizing machine learning, and explored promising future directions for machine learning on POCUS research. The utility of POCUS is largely derived from its capability for real-time inference and portability. While these factors present initial hurdles to the early adoption of machine learning in POCUS, they may also serve as the modalities greatest assets. Machine learning demands increasingly large datasets, sometimes needing millions of training images. POCUS is uniquely positioned to provide large datasets of video frames that could potentially be used for real-time algorithm training. Additionally, the portability of POCUS has the potential to provide a platform for rolling out machine learning applications in medical imaging to the entire world.

## Disclosures

The authors have no conflicts of interest to declare.
